# Mitochondrial Dysfunction and Ion Imbalance in a Rat Model of Hemodialysis-Induced Myocardial Stunning

**DOI:** 10.3390/biomedicines12102402

**Published:** 2024-10-20

**Authors:** Yuxin Nie, Liyu Lin, Qiang Yang, Jiachang Hu, Minmin Sun, Fangfang Xiang, Xuesen Cao, Jinbo Yu, Yaqiong Wang, Jie Teng, Xiaoqiang Ding, Bo Shen, Zhen Zhang

**Affiliations:** 1 Department of Nephrology, Zhongshan Hospital, Fudan University, No. 180 Fenglin Road, Shanghai 200032, China; nie.yuxin@zs-hospital.sh.cn (Y.N.); lin.liyu@zsxmhospital.com (L.L.); 23111210014@m.fudan.edu.cn (Q.Y.); hu.jiachang@zs-hospital.sh.cn (J.H.); xiang.fangfang@zs-hospital.sh.cn (F.X.); cao.xuesen@zs-hospital.sh.cn (X.C.); yu.jinbo@zs-hospital.sh.cn (J.Y.); wang.yaqiong@zs-hospital.sh.cn (Y.W.); teng.jie@zs-hospital.sh.cn (J.T.); ding.xiaoqiang@zs-hospital.sh.cn (X.D.); 2 Shanghai Key Laboratory of Kidney and Blood Purification, No. 180 Fenglin Road, Shanghai 200032, China; 3 Department of Nephrology, Zhongshan Hospital (Xiamen), Fudan University, No. 668 Jinhu Road, Xiamen 361015, China; 4 Nephrology Clinical Quality Control Center of Xiamen, No. 668 Jinhu Road, Xiamen 361015, China; 5 Department of Echocardiography, Zhongshan Hospital, Fudan University, No. 180 Fenglin Road, Shanghai 200032, China; sun.minmin@zs-hospital.sh.cn; 6 Shanghai Institute of Cardiovascular Diseases, Shanghai 200032, China

**Keywords:** myocardial stunning, hemodialysis, mitochondrial dysfunction, chronic kidney disease

## Abstract

**Background/Objectives**: Hemodialysis-induced myocardial stunning (HIMS) is a frequent complication in patients undergoing maintenance hemodialysis, characterized by transient left ventricular dysfunction due to ischemic episodes. Mitochondrial dysfunction and fluctuations in key ions such as potassium (K^+^) and calcium (Ca^2+^) are implicated in the pathogenesis of HIMS. This study aims to investigate the role of mitochondrial dysfunction and the protective potential of mitochondrial ATP-sensitive potassium channels (mitoK_ATP_) in mitigating HIMS. **Methods**: A 5/6 nephrectomy rat model was established to mimic chronic kidney disease and the subsequent HIMS. The effects of mitoK_ATP_ channel modulators were evaluated by administering diazoxide (DZX), a mitoK_ATP_ opener, and 5-hydroxydecanoate (5-HD), a mitoK_ATP_ blocker, before hemodialysis. Mitochondrial function was assessed by measuring membrane potential, ATP synthase activity, and intramitochondrial Ca^2+^ levels. Myocardial function was evaluated using speckle tracking echocardiography. **Results**: Rats undergoing hemodialysis exhibited significant reductions in left ventricular strain and synchrony. DZX administration significantly improved mitochondrial function and reduced myocardial strain compared to controls. Conversely, 5-HD worsened mitochondrial swelling and disrupted myocardial function. Higher K^+^ and Ca^2+^ concentrations in the dialysate were associated with improved mitochondrial energy metabolism and myocardial strain. **Conclusions**: Mitochondrial dysfunction and ion imbalances during hemodialysis are key contributors to HIMS. The activation of mitoK_ATP_ channels provides mitochondrial protection and may serve as a potential therapeutic strategy to mitigate HIMS.

## 1. Introduction

Chronic kidney disease (CKD) is a major global public health challenge that imposes a significant burden on healthcare systems and contributes notably to morbidity and mortality [1]. At its most advanced stage, end-stage renal disease (ESRD), patients must rely on life-sustaining renal replacement therapy (RRT), which includes options such as kidney transplantation or dialysis—primarily hemodialysis and peritoneal dialysis. Hemodialysis is the most widely used treatment modality globally, yet long-term survival rates remain suboptimal [2].

Cardiovascular diseases (CVDs) are disproportionally prevalent in patients undergoing maintenance hemodialysis (MHD), with significantly higher mortality rates compared to the general population, resulting in extremely undesirable survival outcomes [3,4,5].

A variety of well-established cardiovascular therapies often show reduced efficacy or inconclusive benefits in individuals with advanced CKD, particularly in those undergoing dialysis [6,7,8,9]. Likewise, the clinical utility of some conventional biomarkers for prognosis and risk stratification is compromised in this population [10,11], making the management of CVDs in patients with dialysis especially challenging.

A key factor contributing to this complexity is the presence of kidney-specific risks in addition to traditional cardiovascular risk factors such as hypertension, diabetes, and dyslipidemia. These kidney-specific risks include the accumulation of uremic toxins [12,13], CKD-related mineral and bone disorders (CKD-MBDs) [14], and the non-physiological nature of dialysis [15], all collectively contributing to cardiovascular damage, with one notable manifestation being hemodialysis-induced myocardial stunning (HIMS).

HIMS refers to a condition characterized by transient left ventricular systolic dysfunction resulting from ischemic episodes during hemodialysis sessions. With an incidence of approximately 64%, HIMS is common and represents a serious complication associated with the hemodialysis process, contributing to increased cardiovascular morbidity and mortality in this population [16,17,18]. Additional studies confirm that frequent occurrences of myocardial stunning during hemodialysis are linked to adverse cardiovascular outcomes and increased mortality [17,19,20].

Evidence strongly suggests that the hemodialysis process itself, particularly when associated with higher ultrafiltration rates, can lead to myocardial injury through various mechanisms. A reduction in myocardial perfusion is thought to play a key role, resulting in ischemia and subsequent myocardial stunning [16,21]. This phenomenon is marked by an increase in biomarkers of myocardial damage, such as cardiac troponin T and cell-free DNA [21,22,23]. Unfortunately, studies on intra-dialytic intervention are still limited.

Therefore, understanding the pathophysiology of HIMS and developing effective preventive strategies are imperative for improving cardiac function and reducing cardiovascular mortality in patients on MHD.

The myocardium, the most energy-demanding tissue in the body, requires a continuous supply of adenosine triphosphate (ATP) to sustain contractile function. Mitochondria, the principal source of ATP in myocardial cells, are critical to maintaining normal cardiac performance. Mitochondrial dysfunction, characterized by the disruption of ATP synthesis and the opening of mitochondrial permeability transition pores, has been identified as a key contributor to myocardial injury. Consequently, the preservation of mitochondrial integrity is a central focus of cardioprotective strategies [24]. In patients with CKD, mounting evidence indicates that mitochondrial dysfunction is involved in CKD-related cardiovascular complications.

The existence of mitochondrial ATP-sensitive K^+^ channels (mitoK_ATP_) was first reported over two decades ago, sparking significant interest in their role in cellular function [25]. Since then, several mitoK_ATP_ openers, including diazoxide (DZX), nicorandil, BMS191095, and cromakalim, have been identified, and their effects on mitochondrial physiology have been thoroughly explored [26].

It has been established that mitoK_ATP_ play an important role in ischemic preconditioning, positioning them as promising therapeutic targets for cardioprotection during myocardial ischemia. DZX, a mitoK_ATP_ opener with significant selectivity versus the sarcolemmal isoform of the K_ATP_ channel in cardiac myocytes, has been shown to confer protective effects like those of ischemic preconditioning. These protective effects can be blocked by 5-hydroxydecanoate (5-HD), a highly selective K^+^ inhibitor of mitoK_ATP_ [27,28,29]. The activation of mitoK_ATP_ under conditions of ischemia, hypoxia, or energy depletion may preserve mitochondrial integrity and function through the following mechanisms: preserving mitochondrial structure by maintaining osmotic pressure via modulation of K^+^ influx; mitigating Ca^2+^ overload by lowering membrane potential; enhancing mitochondrial respiration [30,31,32].

Therefore, the present study aimed to investigate the role of mitochondrial dysfunction in the onset of HIMS, with a focus on the impact of mitoK_ATP_. The findings could offer new insights into therapeutic interventions for managing cardiovascular risks in patients with CKD on hemodialysis.

## 2. Materials and Methods

### 2.1. Establishment of Animal Model

A 5/6 nephrectomy was performed in two steps, starting with the surgical excision of the upper and lower poles of the left kidney, followed by uninephrectomy on the contralateral side after one week. Throughout the entire study, the rats were on regular chow and had free access to water.

Serum urea and creatinine levels were measured 2, 4, 8, and 12 weeks after operation. The model was considered successfully established if serum creatinine levels were five times higher than baseline.

### 2.2. Vascular Access Creation, Extracorporeal Circulation, and Hemodialysis

Rats that successfully underwent the 5/6 nephrectomy were weighed and anesthetized with an intraperitoneal injection of 2% pentobarbital sodium (3 mg/kg).

The designated areas for catheterization were meticulously shaved and disinfected using 10% povidone–iodine solution. A precise midline incision was made along the anterior aspect of the neck, followed by the careful blunt dissection of subcutaneous and muscle tissues using microvascular clamps and forceps. This dissection facilitated the exposure of the left common carotid artery, where the proximal and distal segments were ligated to secure the vessel. An oblique arteriotomy was then performed on the common carotid artery to insert and secure a PE50 catheter, which provided arterial access for hemodialysis. Using a similar surgical approach, a PE50 catheter was introduced into the left femoral vein. The left common carotid artery and the left femoral vein were utilized as the outflow and inflow routes, respectively.

Following the creation of vascular access, systemic anticoagulation was achieved with an intravenous injection of 500 IU/kg heparin through the femoral vein catheter.

A dialyzer specifically designed for small animals (Wego healthcare, Shandong, China, membrane area: 0.02 m^2^, blood chamber volume: 1.4 mL) was used. The composition and the dialysate were glucose 11 mmol/L, Na^+^ 142 mmol/L, Mg^2^^+^ 1.5 mmol/L, and HCO_3_^−^ 34 mmol/L, with adjustments in K^+^ and Ca^2^^+^ concentrations tailored to the specific requirements of the experimental protocols.

Blood and dialysate flow rates, along with ultrafiltration rates, were meticulously regulated using a small peristaltic pump. The blood flow rate was maintained between 0.8 and 1.0 mL/min, and the dialysate flow rate was set at 0.4–0.5 mL/min. Prior to initiating the hemodialysis, the extracorporeal circulation was primed. Subsequently, the blood pump was activated to initiate blood withdrawal at a rate of 0.3–0.5 mL/min, gradually increasing to the target rate once steady arterial flow was established. Concurrently, the speeds of the replacement fluid infusion pump and the ultrafiltration pump were adjusted to match the set parameters. Throughout the procedure, vital signs such as respiratory rate, heart rate, and blood pressure were rigorously monitored. The limbs were connected to a synchronized electrocardiogram.

**Figure 1:**
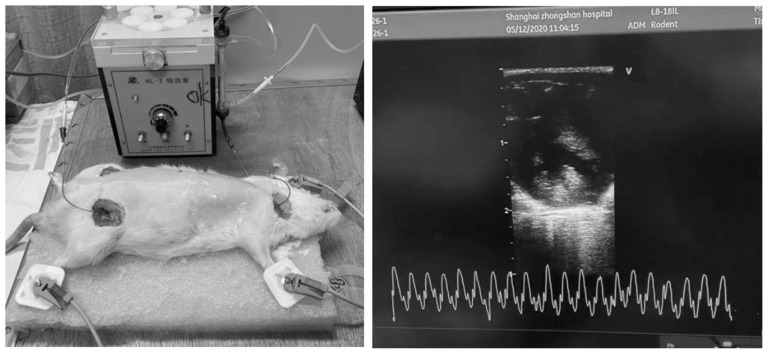
Hemodialysis and echocardiographic monitoring in rats.

Hemodialysis was conducted over a duration of 2 h, with continuous monitoring under anesthesia and systemic anticoagulation using heparin. Following the treatment, extracorporeal circulation was terminated, and the blood within the circuit was safely reinfused into the rats using an air-driven method (Figure 1).

### 2.3. Echocardiography

Echocardiography was performed on the hemodialysis rats prior to dialysis every 30 min during the session and immediately following dialysis, totaling five echocardiographic assessments per session. For these evaluations, rats were positioned supine. Sequential imaging captured three consecutive cardiac cycles across the apical four-chamber, two-chamber, and three-chamber views using a GE Vivid 7 system (GE Medical Systems, Freiburg im Breisgau, Germany). These images were subsequently analyzed offline employing speckle tracking and strain analysis techniques using EchoPAC Clinical Workstation Software (version 113.1, GE, Germany).

### 2.4. Mitochondrial ATP Channel Intervention

To investigate the cardioprotective effects of mitoK_ATP_ modulators, various agents were administered 10 min prior to dialysis. These included the mitoK_ATP_ opener DZX, (3 mg/kg), the mitoK_ATP_ blocker 5-HD (10 mg/kg), a combination of DZX and 5-HD (DZX 3 mg/kg + 5-HD 5 mg/kg), and the calcium channel blocker nicardipine, which was given via intraperitoneal injection (0.4 mg/kg). This pharmacological regimen was designed to delineate the individual and combined effects of these interventions on cardiac function during the stress of dialysis.

### 2.5. Sampling and Processing of Experimental Animals

Upon the completion of dialysis, the heart of each rat was excised along the base of the aorta and immediately immersed in ice-cold saline to halt metabolic processes. After removing adherent impurities and excess fat, residual blood was thoroughly rinsed away. Precise dissections of the left ventricle were performed using sharp blades on a chilled slide, and small samples measuring 1 × 1 mm were excised from the left ventricular myocardium. These samples were promptly fixed in 2.5% glutaraldehyde for subsequent electron microscopy analysis. Additionally, 2–3 pieces of 2 × 2 mm from the ventricular muscle were fixed in 10% formaldehyde for histopathological examination. All remaining animal tissues were disposed of following approved bioethical protocols to ensure safety and compliance with regulatory guidelines.

### 2.6. Grouping of Experimental Animals

Rats were categorized into four groups based on the different K^+^ and Ca^2+^ concentrations in the dialysate: K2 Ca1.25, K2 Ca1.5, K3 Ca1.25, and K3 Ca1.5. After 2 h of hemodialysis, mitochondrial-related tests were conducted, including mitochondrial membrane potential with JC-1, mitochondrial ATP synthase activity, and mitochondrial Ca^2+^ overload.

To investigate the impact of mitochondrial ATP channel intervention, rats were categorized into five groups based on the different drugs: control, diazoxide group, 5-HD group, diazoxide + 5-HD group, and calcium channel blocker group. 

Each group contained four experimental rats.

### 2.7. Statistical Analyses

Data are expressed as mean ± standard error. Between-group comparisons were performed using one-way ANOVA followed by Tukey’s post hoc test. Statistical significance was set at *p* < 0.05. All statistical analyses and visualization were conducted by Rstudio (RStudio 2023.12.1+402 “Ocean Storm” Release, R version 4.4.0).

## 3. Results

### 3.1. Echocardiography

**Figure 2:**
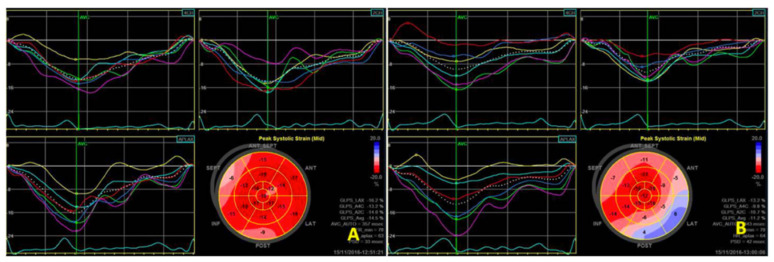
Two-dimensional speckle tracking shows a decrease in left ventricular strain values before (**A**) and immediately after (**B**) dialysis.

Utilizing speckle tracking echocardiography, changes in cardiac function were detected in a hemodialysis rat model. Specifically, the left ventricular multi-segmental strain value (Figure 2) exhibited a marked decrease after dialysis compared to pre-dialysis measurements. The values of longitudinal and circumferential strain at different time points, including those measured during hemodialysis, are detailed in Section 3.3.

### 3.2. Effects of K^+^ and Ca^2+^ Concentrations on Mitochondrial Energy Metabolism and Myocardial Contraction

**Figure 3:**
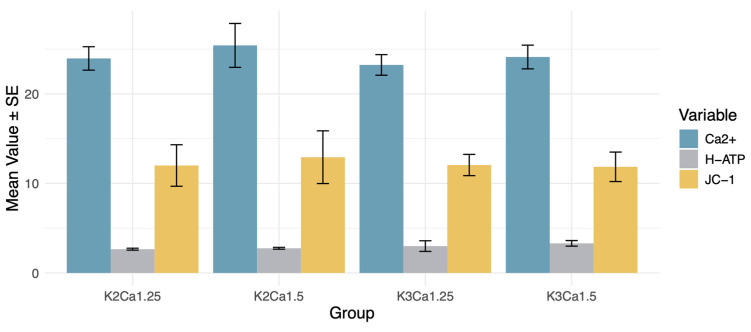
Effects of K^+^ and Ca^2+^ concentrations on mitochondrial energy metabolism. DZX (diazoxide), mitoKATP channel opener; 5HD (5-hydroxydecanoate), mitoKATP channel blocker; CCB (nicardipine), calcium channel blocker; JC-1, mitochondrial membrane potential assay; H-ATP, ATP synthase activity; Ca^2+^, intramitochondrial Ca^2+^ concentration. No between-group differences regarding mitochondrial membrane potential, mitochondrial ATP synthase activity, and mitochondrial Ca^2+^ were observed.

The animals were stratified into four groups based on the potassium and calcium concentrations: K2 Ca1.25, K2 Ca1.5, K3 Ca1.25, and K3 Ca1.5. After 2 h of hemodialysis, mitochondrial function was assessed through measurements of mitochondrial membrane potential, mitochondrial ATP synthase activity, and mitochondrial Ca^2+^ overload. The findings revealed that the group with the highest ion concentrations (K_3_Ca_1_._5_) exhibited a trend toward enhanced ATP synthase activity compared to the group with the lowest ion concentrations (K_2_Ca_1_._25_) (*p* = 0.13). No between-group differences regarding mitochondrial membrane potential, mitochondrial ATP synthase activity, and mitochondrial Ca^2+^ were observed (Figure 3).

### 3.3. Effects of Mitochondrial ATP Channel Modulators and Calcium Channel Blockers on Mitochondrial Energy Metabolism and Myocardial Stunning

Pre-dialysis interventions were administered 10 min before initiation of hemodialysis: the mitoK_ATP_ channel opener DZX (3 mg/kg), the mitoK_ATP_ channel blocker 5-HD (10 mg/kg), a combination of DZX and 5-HD (DZX 3 mg/kg + 5-HD 5 mg/kg), and the calcium channel blocker nicardipine (intraperitoneal 0.4 mg/kg). Declines in both longitudinal and circumferential myocardial strains were observed as hemodialysis progressed, peaking before the end of the session. 

**Figure 4:**
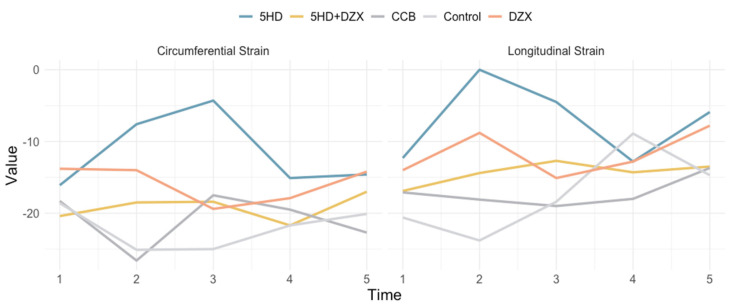
Effects of mitochondrial ATP channel modulators and calcium channel blocking on circumferential strain and longitudinal strain. DZX (diazoxide), mitoKATP channel opener; 5HD (5-hydroxydecanoate), mitoKATP channel blocker; CCB (nicardipine), calcium channel blocker; JC-1, mitochondrial membrane potential assay; H-ATP, ATP synthase activity; Ca^2+^, intramitochondrial Ca^2+^ concentration.

Longitudinal strain (LS) and circumferential strain (CS) of the rat myocardium were analyzed using layer-specific speckle tracking imaging through three-dimensional speckle tracking (Figure 4 and Table 1).

**Figure 5:**
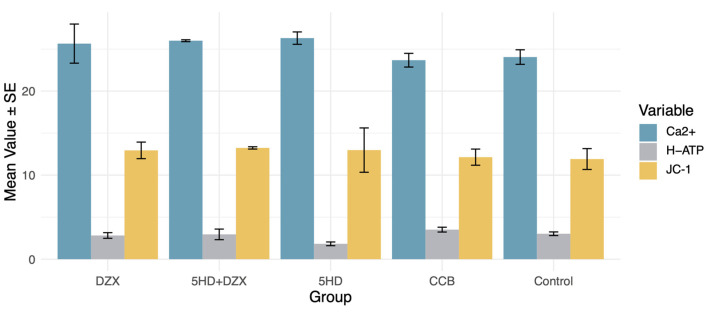
Effects of mitochondrial ATP channel modulators and calcium channel blockers on mitochondrial energy metabolism. DZX (diazoxide), mitoKATP channel opener; 5HD (5-hydroxydecanoate), mitoKATP channel blocker; CCB (nicardipine), calcium channel blocker; JC-1, mitochondrial membrane potential assay; H-ATP, ATP synthase activity; Ca^2+^, intramitochondrial Ca^2+^ concentration.

A similar trend for the harmful impact of 5-HD was also observed in mitochondrial membrane potential, ATP synthase activity, and intramitochondrial Ca^2+^ concentration (Figure 5). However, no significant between-group difference was observed regarding ATP synthase activity (*p* = 0.07), mitochondrial membrane potential (*p* = 0.96), and Ca^2+^ concentration (*p* = 0.44).

**Figure 6:**
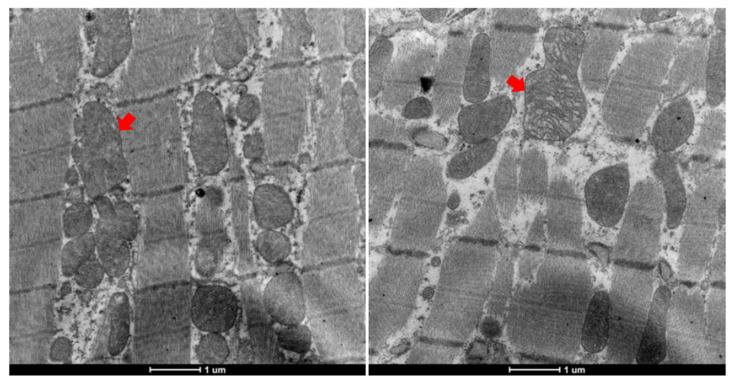
Abnormal mitochondrial morphology in 5-HD-administered rats. (**Left**, mitochondrial swelling; **right**, cristae disruption).

Additionally, rats in the 5-HD group exhibited pronounced mitochondrial swelling, variability in size, and partial cristae disruption (Figure 6).

## 4. Discussion

This study investigated the effects of key ions in the dialysate on mitochondrial function using a 5/6 nephrectomy rat model to simulate the hemodialysis process. Echocardiography showed reductions in both global and segmental contractile functions during and after hemodialysis, accompanied by mitochondrial dysfunction. Intra-dialytic fluctuations in K^+^ and Ca^2+^ also contribute to the onset of myocardial stunning. Activation of mitoK_ATP_ channels exerts a potential protective effect on mitochondrial energy metabolism, highlighting a novel therapeutic target for mitigating hemodialysis-induced cardiac dysfunction.

While larger animals such as swine, dogs, or goats are typically favored for hemodialysis research, our study successfully established a rat model for hemodialysis. Despite certain limitations, such as reduced tolerance to high ultrafiltration, this model provides a robust, accessible, and cost-effective platform to investigate the cardiovascular impacts and underlying mechanisms of hemodialysis.

HIMS is a major contributor to the high incidence of CVD in patients on hemodialysis. Recurrent episodes of myocardial stunning not only impair cardiac function but also have detrimental effects on long-term survival. Unlike traditional ischemia–reperfusion-induced myocardial stunning, which is unpredictable and difficult to manage, HIMS can be anticipated and mitigated given the regular and cyclic nature of hemodialysis treatments and is thus a promising target for intervention. This predictability also positions HIMS as an ideal model for investigating myocardial stunning.

Volume removal is a key objective of hemodialysis, which involves extracting accumulated fluid over 48–72 h within a 4 h session. This rapid volume reduction causes significant hemodynamic shifts that can impair cardiac perfusion and induce myocardial stunning. Research indicates that myocardial blood flow decreases during hemodialysis, which correlates with wall motion abnormalities in regions of reduced blood supply [33,34]. Higher ultrafiltration rates are associated with more severe wall motion abnormalities, particularly in regions supplied by a single vessel [6]. In contrast, peritoneal dialysis, which causes less hemodynamic disturbance, rarely leads to myocardial stunning [35].

Mitochondrial energy metabolism disruption, characterized by ATP depletion, is a crucial factor in myocardial ischemic injury [34]. In hemodialysis animal models, HIMS is associated with mitochondrial ultrastructural damage, including swelling and disordered cristae. Thus, mitochondrial dysfunction from rapid volume reduction during hemodialysis may cause abnormal myocardial contraction. Enhancing mitochondrial energy metabolism could offer a potential strategy for preventing or treating HIMS, with significant clinical implications.

Additionally, solute removal is critical during hemodialysis, particularly for maintaining the balance of key ions like K^+^ and Ca^2+^, which are fundamental for myocardial contractility and rhythm stability. Fluctuations in K^+^ and Ca^2+^ levels during hemodialysis can cause electrophysiological disturbances such as ectopic excitation and conduction abnormalities [36].

K^+^ is essential for maintaining the resting potential of myocardial cells and mitochondria. Our research indicates that the rapid removal of serum K^+^ during dialysis prolongs myocardial repolarization time [37], suggesting that K^+^ fluctuations can affect potassium channel function. Serum potassium imbalances in either direction are problematic in hemodialysis patients. Hyperkalemia is associated with worsened outcomes [38,39], while post-dialysis hypokalemia is associated with an increased risk of ventricular arrhythmias and mortality [40,41]. A multicenter prospective study found that patients dialyzed with a potassium concentration of 1 mEq/L experienced higher mortality rates compared to those using 2 or 3 mEq/L [42]. These findings suggest that milder changes in serum K^+^ concentrations during dialysis may be advantageous.

Drastic intra-dialytic removal of potassium affects cardiac electrophysiology, as the potassium gradient across cell membranes is crucial for repolarization, impacting both resting and action potentials, leading to an increased QT interval, QT dispersion, repolarization heterogeneity, and ultimately arrhythmias [43]. Studies show that profiling potassium levels during dialysis reduces arrhythmic events, with a 36% reduction in premature ventricular complexes observed when profiling is used compared to a constant potassium concentration [44]. Profiling provides smoother potassium clearance than constant low levels, which may decrease arrhythmia risk.

Various potassium channels are present on the inner and outer mitochondrial membranes. During myocardial ischemia, mitochondrial membrane potassium channels open, increasing K^+^ influx, reducing mitochondrial membrane potential, causing membrane depolarization, decreasing mitochondrial Ca^2+^ overload, and promoting mitochondrial respiration. This improves myocardial energy metabolism and protects the ischemic myocardium [45,46]. The rapid decline in serum K^+^ during dialysis prolongs myocardial repolarization time, as rapid cardiac repolarization primarily occurs through K^+^ efflux. This decline potentially impacts potassium channel function. Our results show that rats dialyzed with a higher K^+^ concentration exhibit a trend toward better mitochondrial energy metabolism. We hypothesize that dialysate with a higher K^+^ concentration, by creating a smaller K^+^ gradient between the dialysate and serum, maintains normal mitochondrial potassium channel function and provides mitochondrial protection. Conversely, a larger gradient between the dialysate and serum K^+^ leads to a rapid intra-dialytic decline in serum K^+^, weakening myocardial protection, causing mitochondrial Ca^2+^ overload, disrupting myocardial energy metabolism, and leading to myocardial dysfunction, as indicated by abnormal myocardial strain and contraction synchrony.

Ca^2+^ is the trigger for myocardial cell contraction, and the cyclical changes in cytoplasmic Ca^2+^ concentration during myocardial contraction and relaxation are crucial [47,48]. Also, alterations in intracellular Ca^2+^ regulation are associated with arrhythmia. Abnormal handling of Ca^2+^ in the endoplasmic/sarcoplasmic reticulum could lead to intra-cytoplasmic Ca^2+^ overload and promote arrhythmias such as atrial fibrillation (AF) and ventricular arrhythmias [49]. Intriguingly, the baseline level of sarcoplasmic endoplasmic reticulum calcium ATPase might predict the efficacy of ablative therapy in patients with persistent AF [50].

Hemodialysis patients often suffer from metabolic disorders such as hypocalcemia and hyperphosphatemia, necessitating the use of Ca^2+^-containing dialysate to correct these imbalances. Whether the dialysate Ca^2+^ concentration is 1.5 mmol/L or 1.75 mmol/L, it is usually higher than the serum free Ca^2+^ concentration, leading to Ca^2+^ transfer from the dialysate into the blood, increasing the body’s calcium load. Additionally, chronic malnutrition and hypoalbuminemia in patients on hemodialysis reduce albumin-bound calcium, increasing the proportion of serum free Ca^2+^ and weakening albumin’s buffering capacity against the rapid rise in serum Ca^2+^ during dialysis. Thus, serum Ca^2+^ levels often rise rapidly during dialysis. Intracellular Ca^2+^ overload is a classic mechanism of myocardial cell injury. During myocardial ischemic injury, extracellular Ca^2+^ enters and accumulates in the cytoplasm, and mitochondria take up large amounts of Ca^2+^ to alleviate cytoplasmic Ca^2+^ overload. However, this influx of Ca^2+^ into the mitochondria causes swelling, outer membrane rupture, structural damage, disruption of the electron transport chain, interference with ATP synthesis, and energy metabolism disruption. The rapid intra-dialytic rise in serum free Ca^2+^ during dialysis may exacerbate Ca^2+^ overload in the cytoplasm and mitochondria during ischemia, affecting mitochondrial structure and function, and leading to abnormal myocardial energy metabolism. However, in this study, with fixed dialysate K^+^ concentrations, there was no significant difference in mitochondrial energy metabolism and myocardial strain among the groups with different ion concentrations. During the relatively short dialysis time (2 h in this study) and low dialysate flow rate, intracellular calcium fluctuations in myocardial cells were not as drastic as expected. It should be noted that calcium channel blocker usage displayed a trend toward cardiac protection. Future studies using larger animals with longer dialysis times and a higher dialysate flow rate may provide more conclusive findings.

In clinical settings, the dialysate Ca^2+^ is a modifiable factor that presents a therapeutic dilemma. Higher dialysate Ca^2+^ levels (1.75 mmol/L) may help manage low PTH and prevent hypercalcemia but may exacerbate vascular calcification and stiffness, contributing to cardiovascular complications. Lower dialysate Ca^2+^ concentrations have been reported to increase QT dispersion and increase the risk of arrythmia [51,52].

HIMS is fundamentally a mild and transient myocardial ischemic injury. Ischemic preconditioning is one of the most effective strategies for preventing ischemic injury [27,53,54]. Previous studies have identified mitoK_ATP_ as critical mediators in ischemic preconditioning. DZX, a specific mitoK_ATP_ opener, has demonstrated protective effects similar to those of ischemic preconditioning [27,28].

In the present study, DZX significantly reduced mitochondrial Ca^2+^ levels compared to the control and 5-HD groups, indicating that DZX alleviates Ca^2+^ overload by promoting mitoK_ATP_ channel opening. This protective effect was reversed by mitoK_ATP_ blockage. Additionally, echocardiographic assessments showed less severe myocardial strain reduction during dialysis in DZX-treated rats. Electron microscopy revealed marked mitochondrial swelling and structural damage in the 5-HD group.

These findings underscore the critical role of mitoK_ATP_ channels in maintaining mitochondrial structure and energy metabolism, with mitoK_ATP_ activation offering potential protection against ischemic myocardial injury. While DZX’s cardioprotective effects have been established in ischemia–reperfusion models, the unpredictable nature of traditional myocardial stunning complicates precise prevention. In contrast, the scheduled and cyclical nature of hemodialysis allows for the accurate prediction of HIMS onset, facilitating targeted pharmacological intervention.

Several limitations should be noted. First, this study utilized a rat model, and extending the dialysis duration to a standard 4 h was not feasible due to the physiological constraints of the animal. This limited our ability to fully assess the effects of the routine clinical four-hour hemodialysis. It should also be noted that rapid ultrafiltration is another paramount risk factor for CVD during hemodialysis. But, due to the challenges of ensuring stable extracorporeal circulation in rats, the ultrafiltration was minimal. Validation using large animal models would provide a more comprehensive investigation, taking into consideration both the solutes and fluid removal.

## 5. Conclusions

Mitochondrial dysfunction induced by myocardial ischemia during hemodialysis is a crucial factor in the development of HIMS, leading to impaired myocardial contractility. Intra-dialytic fluctuations in K^+^ and Ca^2+^ also contribute to the onset of myocardial stunning. Activation of mitoK_ATP_ channels exerts a protective effect on mitochondrial energy metabolism, suggesting that exogenous promotion of mitoK_ATP_ channel opening may offer potential therapeutic protection against HIMS.

## Figures and Tables

**Table 1:** Longitudinal and circumferential strain measurements across experimental groups over time.

Group	Pre-Dialysis		30 Min		60 Min		90 Min		Post-Dialysis	
	CS	LS	CS	LS	CS	LS	CS	LS	CS	LS
DZX	−13.8	−14	−14	−8.8	−19.4	−15.1	−17.9	−12.8	−14.2	−7.8
DZX + 5HD	−20.4	−16.9	−18.5	−14.4	−18.4	−12.7	−21.7	−14.3	−17	−13.5
5HD	−16.1	−12.3	−7.6	0	−4.3	−4.5	−15.1	−12.8	−14.6	−5.9
CCB	−18.3	−17.1	−26.6	−18.1	−17.5	−19	−19.5	−18	−22.7	−13.7
Control	−18.6	−20.6	−25.1	−23.8	−25	−18.4	−21.7	−8.9	−20.1	−14.7

DZX (diazoxide), mitoKATP channel opener; 5HD (5-hydroxydecanoate), mitoKATP channel blocker; CCB (nicardipine), calcium channel blocker; CS, circumferential strain, presented as %; LS, longitudinal strain, presented as %.

## Data Availability

The data presented in this study are available on request from the corresponding author.
